# The effect of social activities on the alienation and family resilience of Chinese caregivers for children with autism: a latent class analysis

**DOI:** 10.3389/fpsyt.2024.1406073

**Published:** 2024-06-04

**Authors:** Lin Zheng, Chen Long, Wongyu Choi

**Affiliations:** ^1^ Department of Social Welfare, Jeonbuk National University, Jeonbuk, Republic of Korea; ^2^ Health Services Management Department, Guizhou Medical University, Guiyang, China

**Keywords:** Chinese caregivers for children with autism, social activities, alienation, family resilience, latent class analysis

## Abstract

**Introduction:**

Caregivers of children with autism spectrum disorder (ASD) in China often experience alienation due to societal stigma. While this alienation detrimentally impacts their mental well-being, family resilience serves as a protective factor. Previous research has predominantly examined the social support derived from social activities but has neglected to delve into the specific patterns of these activities. The primary objective of this study was twofold: firstly, to gain insights into the various social activities engaged in by caregivers of children with autism in China, and secondly, to ascertain the influence of these social activities on alienation and family resilience.

**Methods:**

Between June and August 2023, a cross-sectional survey was carried out across multiple cities in Jilin Province, aiming to gather data from a total of 205 Chinese caregivers of children with autism. Data collection was conducted through the utilization of a structured questionnaire. The assessment of social activity involved the completion of 12 questionnaires, while alienation was evaluated using the Generalized Alienation Scale (GSAS), and family resilience was gauged through the Chinese version of the Family Resilience Scale (FaRE). The classification of social activities was conducted through latent class analysis (LCA), while the impact of these social activities on alienation and family resilience was examined using linear regression analysis.

**Results:**

The findings revealed that social activities can be categorized into five types (Low, Self-Recreation, Communication, Web Surfing, High). Communication social activities were found to reduce family resilience(β=.332, p<0.01), while high social activities were associated with reduced alienation(β=-.349, p<0.05) and increased family resilience(β=.417, p<0.01).

**Conclusion:**

Supporting these particular types of social activities has the potential to reduce alienation and bolster family resilience among caregivers for children with autism in China.

## 1 Introduction

Recently, there has been a persistent rise in the prevalence of diagnosed autism among children globally. According to the latest data from the Centers for Disease Control and Prevention’s (CDC) Morbidity and Mortality Weekly Report (MMWR), a statistical analysis for the year 2020 indicates that 1 in 36 (2.76%) 8-year-olds are diagnosed with an autism spectrum disorder (ASD). This figure is higher than the reported value in 2018 (2.3%) (1). The escalating number of children with autism has led to an increased number of families caring for them, emphasizing the pressing need to address caregiver-related challenges (2). Many caregivers, tasked with caring for a family member with autism, grapple with personal burden and shortened work hours, significantly impacting their lives (3–5).

Caregivers of children with autism not only bear the responsibility themselves but are also subjected to societal stigma due to their role (6–8). Stigma, defined as “an attribute that is deeply discrediting,” signifies “undesired differentness” (9). In China, caregivers of children with autism face the stigma associated with the condition, and this issue remains inadequately addressed. This not only affects their quality of life (10), but also leads to their ostracization by society and gradual alienation (11, 12). Although societal stigmatization inflicts harm, caregivers internalize negative stereotypes from the public. They continue to assimilate these stereotypes into their psychological identity, thereby impacting their mental well-being (13, 14). Throughout this process of stigmatization, caregivers of children with autism further exacerbate their sense of alienation and become increasingly marginalized in society (15, 16). Research indicates that alienation significantly exacerbates mental health issues, particularly depression (17, 18). The majority of research concerning caregivers of children with autism directly addresses their physical health. However, initial stigmatization precedes alienation, with subsequent ramifications including more severe issues like depression or suicide. Consequently, this study advocates for increased focus on the alienation experienced by Chinese caregivers of children with autism.

Amidst these experiences of alienation, families dealing with autism encounter challenges while also experiencing growth. Research on families with autism reveals that family resilience enables them to mitigate parental stress and enhance life satisfaction (19, 20). Despite varying levels of family resilience among caregivers of children with autism, its protective significance underscores the ongoing research on family resilience within these families (21, 22). Whereas caregivers of children with autism often experience alienation and health decline, their family resilience empowers them to confront challenges and alleviate stress. These two variables emerged as primary factors in assessing caregivers of children with autism in this study.

The majority of research within the social sciences concerning caregivers of families with autism has been directed towards investigating the dynamics of autism family social support. The act of seeking social support serves as a crucial mechanism through which caregivers can subjectively navigate their circumstances (23–25). Nevertheless, the majority of these studies, which focus on the social support sought by caregivers, have predominantly provided descriptive accounts of its outcomes. The specifics of social activities engaged in during the pursuit of social support have not been thoroughly examined. Effective social support significantly influences the well-being and resilience of caregivers of children with autism (26). Engagement in social activities is intricately linked to feelings of loneliness as well as to both physical and mental well-being (27, 28). This study aims to categorize the daily social activities undertaken by caregivers and to conduct a comprehensive analysis of how various social activities impact the alienation and family resilience of caregivers of children with autism.

This study aims to achieve two primary objectives. Firstly, it seeks to develop a typology of social activity groups among caregivers of children with autism through Latent Class Analysis (LCA) and to discern disparities and commonalities within the empirically derived groups. Secondly, it endeavors to investigate the significance of these groups on the experiences of alienation and the resilience of families caring for children with autism. The examination of LCA, alienation, and family resilience within the realm of social activities among Chinese caregivers of children with autism holds considerable importance. It promises to offer insights into the influence of social activities on the prevalent sense of alienation experienced by this demographic and their contribution to family resilience. The findings are anticipated to inform the development of more effective social activities tailored to their needs, thereby enhancing their access to social support.

## 2 Materials and methods

### 2.1 Data

Given the potential correlation between the phenomenon of left-behind children in China and the incidence of autism in children (29), caregivers who were not residing with their children for extended durations were excluded from our research cohort. Consequently, this survey specifically targeted caregivers with over three months of caregiving experience for children diagnosed with autism. In this study, 205 Chinese caregivers caring for children with autism were surveyed between June and August 2023.

The survey primarily targeted the provincial area of Jilin Province, China. Its primary objective was to assess the mental health status of caregivers of children with autism and examine the dynamics within their families. Ethical clearance for the study was obtained from the Biomedical Ethics Review Board of Jeonbuk National University (IRB2023-06-003-002).

Surveys were administered independently by a sole investigator, with each autistic child’s family represented by a caregiver participant, ensuring the reliability of the survey sample. The completion of each questionnaire typically requires between 10 to 15 minutes. Researchers initiated contact with administrators of various communities, foundations, and organizations frequented by autism caregivers. With their assistance, researchers secured consent from caregivers of autistic children to participate in the questionnaire. During questionnaire completion, researchers remain available to address any queries from the caregivers. To uphold participant confidentiality, responses in the questionnaire are coded to prevent direct identification of respondents.

### 2.2 Measures

#### 2.2.1 Dependent variables

The assessment of alienation utilized the Generalized Social Alienation Scale (GSAS) developed by Jessor R & Jessor S (30). This scale, adapted for use in Chinese social research, particularly among youth and older adults, has demonstrated robust reliability and validity within the Chinese population (31, 32). Comprising 15 questions across four dimensions—alienation from others, skepticism, self-alienation, and meaninglessness—participants rated their perceptions on a four-point Likert scale (1=Strongly Disagree; 2=Disagree; 3=Agree; 4=Strongly Agree). The scores for the fifteen questioned items were added together and averaged, which is the score for feelings of alienation (On a scale of 1 to 4). Higher scores indicate higher levels of alienation. The Cronbach’s alpha for feelings of alienation was 0.936. The Cronbach’s alpha coefficient for this assessment was 0.936. Distribution analysis revealed skewness at 0.002 and kurtosis at 2.073, confirming adherence to a normal distribution pattern.

In addition, family resilience was gauged using the Family Resilience Scale (FaRE) developed by Faccio et al. (33). And the Chinese version of FaRE Questionnaire has acceptable reliability and validity among patients with breast cancer in China (34). The assessment of family resilience consists of 24 questions and is composed of 4 factors: communication and cohesion, perceived social support, religiousness and spirituality and eigenvalues. Family resilience was measured on the same 4-point Likert scale as the alienation measure (1=Strongly Disagree; 2=Disagree; 3=Agree; 4=Strongly Agree). The average of the 24 questioned items is the score for family elasticity. (On a scale of 1 to 4). Higher scores indicate higher levels of family resilience. The Cronbach’s alpha for feelings of alienation was 0.935. The skewness of family resilience was -0.668. kurtosis at 3.256. Again, this conforms to a normal distribution.

#### 2.2.2 Independent variables

The independent variable in this study is the type of social activities. These activities encompassed 12 questions, with the initial ten drawn from the ‘China Health and Retirement Longitudinal Study (CHARLS)’ survey (35). The remaining two questions pertained to self-learning and interacting with other families with ASD. **Table 1** displays the 12 items comprising these social activities, as employed in our investigation. For the latent class analysis (LCA), the social activities were dichotomized in **Table 1** (0=not experienced, 1=experienced).

**Table 1:** Content of the social activity questionnaire.

Items	Questionnaire
Interact with friends	Visiting and socializing with friends.
Free time	Playing Ma-jong, chess, or cards, or going to a community club.
Informal aid	Providing help to family, friends, or neighbors who do not live with you.
Sports	Dancing, engaging in fitness activities, and practicing Qigong.
Community group	Participating in activities organized by associations.
Volunteer work	Volunteering for activities or charity events.
Care	Caring for a sick or disabled person who does not live with you.
Educational	Going to school or attending a training program.
Stock investment	Speculating in stocks.
Internet	Surfing the internet.
Self-learning	Reading books, newspapers, or magazines.
Network	Gathering with other parents and attending meetings.

#### 2.2.3 Covariate variables

Covariate variables were chosen based on prior research examining factors influencing the alienation and family resilience of caregivers of individuals with autism. The selected variables encompassed gender (male, female), age categories (Youth, Middle-aged, Elderly), average monthly income (in yuan), educational attainment (<=Middle school, >=High school), and presence of chronic disease (yes, no) (36–39). Among these variables, age classification follows the United Nations World Health Organization’s guidelines: individuals under 44 are categorized as younger adults, those aged 44 to 59 as middle-aged adults, and those 60 and older as older adults (40).

#### 2.2.4 Statistical analysis

Latent Class Analysis (LCA) was utilized to categorize social activities into discrete groups. LCA, recognized as a person-centered analytical method for discerning exposure patterns, facilitates the grouping of participants exhibiting comparable response patterns into distinct classes. We determined that employing latent class analysis for type discrimination would better capture the actual participation of Chinese caregivers of children with autism in social activities. Hence, this approach was adopted for the study.

This study aimed to investigate the impact of social activities on the health outcomes of caregivers of children in China. To achieve this, we employed a structured three-step analytical methodology. Initially, we performed a descriptive analysis to characterize the study cohort, summarizing relevant demographic and clinical variables. Subsequently, Latent Class Analysis (LCA) was utilized to identify distinct groups of social activities, each named based on its unique characteristics. Finally, regression analysis was conducted to evaluate the influence of latent classes of social activities on alienation and family resilience. This comprehensive analytical approach allowed for a thorough exploration of our research questions, providing valuable insights into the role of latent classes of ACEs in the health outcomes of caregivers of children. Data analysis utilized the Mplus 8.3 program for LCA and Stata 17.0 for multiple regression analyses.

## 3 Results

### 3.1 Descriptive statistics

The basic information of the sample is presented in the first column of **Table 2**, summarizing sociodemographic characteristics. The sample exhibits a balanced gender distribution, with 19.51% males and 80.49% females. Accordingly, 60.98% are younger adults, 25.37% are middle-aged adults, and 13.66% are elderly. The average monthly income of caregivers is 3,963.415 RMB (SD=3657.503). Educational attainment is divided into two groups based on interviews, considering that older adults caregivers tend to have lower education levels. The groups are middle school and below, and above middle school, with 48.78% having education below middle school. Regarding chronic disease, the majority (72.20%) did not report any chronic illness in the past six months.

**Table 2:** Associations between variables and social activities groups.

	Total (n=205)	Low	Self-Recreation	Communication	Web Surfing	High	F/X²
Gender							
Male	40 (19.51%)	3	6	7	10	14	2.637*
Female	165 (80.49%)	40	5	52	45	23	
Age							
Young	125 (60.98%)	23	1	40	37	24	3.10* (a>b>c)
Middle-aged	52 (25.37%)	15	0	13	13	11	
Old	28 (13.66%)	5	10	6	5	2	
Average monthly income(yuan)	3963.415 (3657.503)	3393.023 (2587.552)	4072.727 (1047.942)	3971.186 (4680.924)	3254.545 (2987.556)	5635.135 (3868.693)	135.010
Education							
<=Middle school	100 (48.78%)	27	9	29	21	14	1.615
>=High school	105 (51.22%)	16	2	30	34	23	
Chronic Disease							
Yes	57 (27.80%)	16	10	11	13	7	-.707
No	148 (72.20%)	27	1	48	42	30	
Alienation	2.368 (.654)	2.564 (.612)	2.455 (.830)	2.316 (.521)	2.493 (.662)	2.013 (.705)	178.260
Family resilience	2.741 (.604)	2.501 (.708)	2.662 (.551)	2.877 (.440)	2.601 (.570)	3.033 (.617)	233.651

Young=a, Middle-aged=b, Old=c. *p<.05.

The findings in **Table 2** encompass outcomes regarding alienation and family resilience variables. Across the five social activity groups, the mean alienation score was 2.368 (range: 1-4), and the mean family resilience score was 2.741 (range: 1-4). Notably, the low social activity group exhibited the highest alienation scores (mean = 2.564) and the lowest family resilience scores (mean = 2.013). Conversely, the high social activity group displayed the lowest alienation score (mean = 2.501) and the highest family resilience score (mean = 3.033).

### 3.2 Types of social activities

This study employed latent class analysis for type discrimination, chosen for its ability to better reflect the actual social activity participation patterns among guardians of children with autism in China.

**Table 3** presents the model fit statistics for each evaluated model. The optimal class model was selected based on model fit indices, including AIC, BIC, aBIC, Entropy, and LMR-LRT, commonly used in such analyses, to determine the social activity class model. As indicated in **Table 1**, the five-group latent class models exhibited the lowest AIC (2518.475) and aBIC (2528.374) values, along with the highest Entropy value (0.750). Additionally, the LMR-LRT values were statistically significant (p<.05). These findings suggest that the five-group latent class model provides the most consistent description of social activity types.

**Table 3:** Fitness of latent class analysis model.

		Latent Class Model					
		2	3	4	5	6	
Information Index	AIC	2567.361	2551.621	2525.321	**2518.475**	2531.249
	BIC	2650.436	2677.895	2694.795	2731.148	2787.121
	aBIC	2571.228	2557.498	2533.209	**2528.374**	2543.158
Model Comparison	BLRT	.000	.000	.050	.030	1.000
Quality of Classification	Entropy	.554	.644	.729	**.750**	.749
Classification Ratio (%)	1	113(55.1)	56(27.3)	11(5.4)	43(21.0)	11(5.4)
	2	92(44.9)	49(23.9)	64(31.2)	11(5.4)	9(4.4)
	3		100(48.8)	56(27.3)	59(28.8)	42(20.5)
	4			74(36.1)	55(26.8)	31(15.1)
	5				37(18.0)	52(25.4)
	6					60(29.3)

Values in bold are the corresponding optimal values.

**Figure 1:**
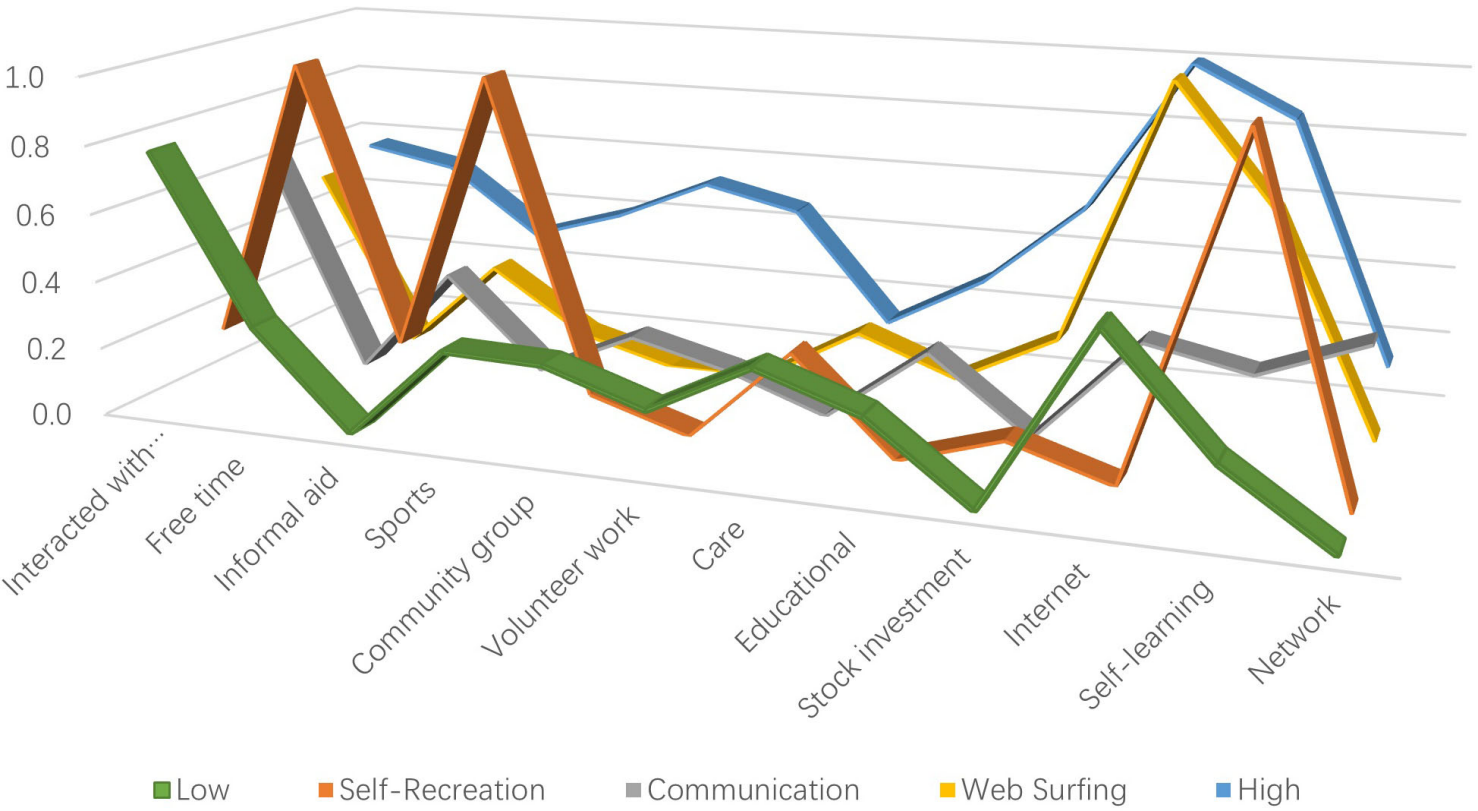
Conditional probability of social activities.

As illustrated in **Figure 1** and **Table 4**, Group 1 and Group 3 exhibited relatively low probabilities across the 12 social activity questions, with Group 3 showing higher probabilities for interactions with friends, informal assistance, and networking. These activities predominantly involve interpersonal communication, leading to the designation of Group 3 as “Communication.” Group 1, characterized by lower probabilities across all questions except for interactions with friends, was consequently labeled “Low”.

**Table 4:** Estimation of probabilities of social activities groups.

	Low	Self-Recreation	Communication	Web Surfing	High
Interacted with friends	.779	.168	.629	.523	.568
Free time	.286	1.000	.000	.002	.515
Informal aid	.000	.186	.316	.266	.310
Sports	.275	1.000	.046	.078	.397
Community group	.267	.091	.178	.000	.523
Volunteer work	.171	.000	.106	.000	.458
Care	.323	.278	.000	.169	.122
Educational	.223	.000	.223	.059	.284
Stock investment	.000	.093	.000	.210	.536
Internet	.527	.000	.315	1.000	1.000
Self-learning	.203	1.000	.254	.640	.852
Network	.000	.000	.371	.000	.133

Group 2 demonstrated the highest probabilities for leisure time, sports, and self-learning, indicating a focus on recreational activities. Therefore, it was termed “Self-Recreation”.

Both Group 4 and Group 5 displayed higher probabilities for networking and self-learning activities. However, while Group 4 primarily engaged in these two activities, its involvement in other social activities was generally lower compared to the other groups, leading to the designation of “Web Surfing.” In contrast, Group 5 exhibited higher probabilities of social activity across most of the 12 questions, earning them the label “High”.

Among the five groups, the third group (n=59) and the fourth group (n=55) accounted for 28.8% and 26.8% of the total sample, respectively, representing the largest proportions. In contrast, the second group (n=11) comprised only 5.4% of the total sample, making it the group with the lowest proportion. Finally, Group 1 (n=43) constituted 21.0% of the total sample, while Group 5 (n=37) accounted for 18.0%.

As shown in **Table 2**, we examined the diversity in sociodemographic factors among the five sub-groups. The t-test analysis demonstrated that female caregivers engaged in a greater number of social activities compared to their male counterparts. Additionally, the chi-square test results indicated that younger caregivers participated in more social activities than their middle-aged and older counterparts.

### 3.3 Multiple regression analysis

**Table 5** shows the model fit indicators F-statistic and adj. R². In the alienation model, the F-value was 5.29 (p<.001), and the adjusted R² was 0.174, indicating that the fitted model accounts for 17.4% of the variance in alienation scores. Similarly, in the context of the family resilience model, the F-statistic registers at 4.58 (p<.001); the adjusted R² was 0.149, suggesting that the fitted model explains 14.9% of the variability in family resilience scores.

**Table 5:** Multiple regression analysis on alienation and family resilience.

	Alienation		Family resilience	
	β	SE	β	SE
Latent class (ref. Low)				
Self-Recreation	-.262	.240	.072	.225
Communication	-.151	.122	.332**	.114
Web Surfing	.003	.125	.062	.117
High	-.349*	.142	.417**	.133
Gender	.094	.114	-.137	.107
Age				
Middle-aged	-.111	.099	.190*	.093
Old	-.050	.148	.285*	.139
Average monthly income(yuan)	-.000**	.000	.000	.000
Education	-.129	.089	.085	.084
Chronic Disease	-.369***	.101	.242*	.094
Constant	2.927***		2.291***	
R²	.214		.191	
adj. R²	.174		.149	
F	5.29***		4.58***	

*p<.05, **p<.01, ***p<.001.

After controlling for demographic variables, a significantly associated sense of alienation was found in the high social activity group compared to the low social activity group (β=-.349, p<.05). Also, the variables of chronic disease presence (β=-.369, p<.001) and average monthly income (β=-.000, p<.01) were significantly associated with feelings of alienation. While in the family resilience model, the communication social activity group (β=.332, p<.01) and the high social activity group (β=417, p<.01) were significantly associated with alienation compared to the low social activity group.

Average monthly income was no longer significantly compared to the alienation model, but the variable of chronic disease presence remained significantly (β=.242, p<.05). Also seen in the variable of age was higher family resilience for middle-aged adults (β=190, p<.05) and older adults (β=285, p<.05) caregivers compared to young caregivers.

## 4 Discussions

This study utilized Latent Class Analysis (LCA) to estimate the effects of various groups—Low, Self-Recreation, Communication, Web Surfing, and High—on alienation and family resilience among Chinese caregivers of children with autism. The findings suggest that the social activities in which caregivers engage significantly impact their sense of alienation and family resilience, even after controlling for sociodemographic variables. Specifically, participants involved in communicative social activities demonstrated a positive influence on family resilience compared to other groups. Conversely, those engaged in high levels of social activity showed a negative impact on alienation and a positive effect on family resilience. This is further evidence that communicative social activities and a high frequency of social events can help caregivers of children with autism to feel less alienated and increase their family resilience.

Prior research has highlighted the impact of various social activities, such as those discussed by Lee and Kim (41) on mental health, particularly depression, and by Kim and Kang (42) on life satisfaction. However, these studies have predominantly focused on the frequency and quality of social engagements, neglecting the specific content of such activities. In contrast, our study adopts a person-centered approach rather than a variable-centered one to analyze social activity. By doing so, we contribute to the identification of latent groups or patterns of social engagement concealed within the data, thereby enhancing our understanding of the heterogeneity within the study population. Notably, social activity among caregivers of children with autism has received scant attention. Despite advocating for increased social support, little research has delved into the nature of the social activities these caregivers engage in daily. Our study aims to address this gap in the literature.

This study revealed a significant association between the caregivers’ chronic disease status and their experiences of alienation as well as family resilience while caring for children with autism. Rearing a child with autism spectrum disorder constitutes a substantial chronic stressor capable of disrupting multiple aspects of adult life and precipitating mental health issues. Consequently, greater attention should be devoted to their well-being, as it directly influences their mental health and resilience development.

In this study, Latent Class Analysis (LCA) was employed to evaluate the impact of different social activity groups on alienation and family resilience among Chinese caregivers of children with autism. Specifically, the type of social activities that focus on high-frequency and communicative social activities are effective in increasing family resilience, while high-frequency social activities allow them to reduce feelings of alienation and protect mental health. As introduced, caregivers of children with autism encounter stigma in their lives, setting them apart from other caregivers in society and fostering a sense of alienation. Nevertheless, in accordance with resilience theory, they actively strive to surmount challenges and enhance their quality of life amidst adversity (43). The caregivers of children with autism participating in this study engage in social activities, particularly those involving communication and high-level social interaction, which contribute significantly to diminishing their alienation and enhancing their family resilience over time. It is crucial to acknowledge the efforts exerted by caregivers of children with autism, especially amidst prevalent stigmatization and the emerging characterization of this group (44). Consequently, assisting them in shedding labels and fostering integration into society becomes imperative. Communities and healthcare providers interacting with caregivers of children with autism should integrate engagement in high-level and communication-based social activities and services. This holistic approach aims to facilitate greater participation and enjoyment among caregivers of children with autism. Moreover, the findings of this study can offer valuable theoretical underpinnings for the future design of social support programs tailored to this demographic.

This study faces several limitations. Firstly, the surveyed sample was confined to Jilin Province, China, lacking geographical diversity. Secondly, linguistic misunderstandings hampered cross-cultural generalizability during variable interrogation, thus failing to fully encapsulate cultural nuances in the results. Thirdly, despite drawing from pertinent research, the design of social activity inquiries lacked theoretical rationale and comprehensive understanding of caregivers’ engagement with autistic children. Future research should adopt a more systematic approach to social activity variables.

## 5 Conclusion

This research investigated the diversity of social activity types and their association with feelings of alienation and family resilience among a cohort of Chinese caregivers of autistic children. The results indicate that social activities tend to coalesce into identifiable clusters, facilitating categorization. Consequently, targeted implementation of such activities holds promise for mitigating alienation symptoms and enhancing family resilience within this demographic. Notably, prioritizing high-frequency and communication-centric social activities in subsequent interventions may yield optimal outcomes. Such a focused approach has the potential to significantly alleviate alienation and counteract stigma experienced by caregivers for children with autism.

## Data availability statement

The raw data supporting the conclusions of this article will be made available by the authors, without undue reservation.

## Ethics statement

The studies involving human participants were reviewed and approved by the Biomedical Ethics Review Board of Jeonbuk National University (IRB2023-06-003-002). The patients/participants or patients/participant’s next of kin provided their written informed consent to participate in this study. Written informed consent was obtained from the individual(s) for the publication of any potentially identifiable images or data included in this article.

## Author contributions

LZ: Conceptualization, Data curation, Formal analysis, Software, Validation, Writing – original draft, Writing – review & editing. CL: Conceptualization, Data curation, Writing – original draft, Writing – review & editing. WC: Funding acquisition, Methodology, Project administration, Supervision, Validation, Writing – review & editing.
